# Global research trends and hotspots on environmental pollution and primary liver cancer: a bibliometric and visualized analysis

**DOI:** 10.1186/s41182-025-00820-7

**Published:** 2025-10-30

**Authors:** Jingqin Hu, Xue Jiao, Yuchang Wang, Huiwen Yang, Xiaohan Zhang, Feng Jiang, Ping Li

**Affiliations:** 1 The Second People’s Hospital Affiliated to Tianjin Medical University, Tianjin Medical University, Tianjin, China; 2 Department of Hepatology, Tianjin Second People’s Hospital, Tianjin, 300192 China; 3 Tianjin Research Institute of Liver Diseases, Tianjin, China; 4 Tianjin Medical University General Hospital, Tianjin Medical University, Tianjin, China; 5 Second Hospital of Tianjin Medical University, Tianjin Medical University, Tianjin, China; 6 College of Integrated Traditional Chinese and Western Medicine, Tianjin University of Traditional Chinese Medicine, Tianjin, China; 7 Department of Neonatology, Obstetrics & Gynecology Hospital of Fudan University, Shanghai Key Lab of Reproduction and Development, Shanghai Key Lab of Female Reproductive Endocrine Related Diseases, Shanghai, China

**Keywords:** Primary liver cancer, Environmental pollution, Bibliometric analysis, CiteSpace

## Abstract

**Background:**

Primary liver cancer (PLC) ranks as the third leading cause of cancer-related mortality worldwide, posing a serious global public health burden. Hepatocellular carcinoma (HCC) is the most common subtype, accounting for approximately 75%–85% of all PLC cases. In recent years, environmental pollution has emerged as a potential risk factor for PLC. However, a systematic bibliometric analysis of global research trends in this field remains lacking. This study aims to perform a comprehensive bibliometric analysis to explore global trends in the field of environmental pollution and PLC research from 2000 to 2025.

**Methods:**

We conducted a bibliometric analysis using the Web of Science Core Collection database, covering studies published from January 2000 to March 2025. CiteSpace was used to analyze publication trends, global collaborations, and key research areas through network visualizations and co-occurrence analyses.

**Results:**

A total of 562 publications were included in this study, with China and the United States identified as the leading contributors. Prominent institutions in this field include the National Institutes of Health (USA), National Cancer Institute (USA), and the Chinese Academy of Sciences. Among journals, Environmental Health Perspectives, Hepatology, and Cancer Research were the most frequently cited, indicating a close connection between environmental science and oncology. Keyword analysis revealed that research focuses not only on traditional pollutants such as air pollution and heavy metals, but also on emerging exposures including volatile organic compounds and drinking water contaminants. Mechanistic studies remain at the core of this field, with frequently occurring terms such as “oxidative stress”, “gene expression”, and “inflammation”. Meanwhile, clinical research-related keywords like “epidemiology” and “follow-up” have become increasingly prominent in recent years, indicating a growing emphasis on population-based risk assessment.

**Conclusions:**

This study highlights the growing research interest in the link between environmental pollution and PLC. Cross-disciplinary collaborations between environmental science, medicine, and public health are increasingly influencing the development of this field. Future research should focus on elucidating the carcinogenic mechanisms of pollutants and enhancing translational applications in public health.

## Introduction

Primary liver cancer (PLC) ranks as the third leading cause of cancer-related mortality worldwide, with approximately 865,000 new cases diagnosed globally in 2022 [1]. At the national level, Mongolia reports the highest incidence of primary liver cancer worldwide, with an age-standardized incidence rate of approximately 94 per 100,000 [2]. Among PLC subtypes, hepatocellular carcinoma (HCC) accounts for 75%–85% of cases, followed by cholangiocarcinoma (CCA), which comprises 10% to 15% [3]. Rarer forms, such as hepatic angiosarcoma and pediatric hepatoblastoma, constitute only a small proportion. Despite being the sixth most commonly diagnosed malignancy, PLC is associated with a dismal 5-year survival rate of just 18% [4]. Although recent advances in surgical resection, liver transplantation, and systemic therapies have improved outcomes to some extent, the overall prognosis for PLC remains poor [5]. Traditional surveillance methods, including ultrasonography and alpha-fetoprotein (AFP) testing, lack sufficient sensitivity and specificity for early HCC detection [6]. Consequently, most patients are diagnosed at an advanced stage, when curative treatment options are limited, and the median survival for advanced liver cancer is only 6–8 months [7]. These challenges highlight the critical need to improve early detection and to address modifiable risk factors. In previous studies, the etiology of PLC has primarily focused on liver-specific diseases, including chronic hepatitis B and C infections, alcoholic liver disease, and metabolism-associated fatty liver disease (MAFLD) [8]. Recently, increasing attention has been given to environmental pollution, which may play a pivotal role in PLC development and could offer promising avenues for prevention.

An increasing body of evidence suggests that environmental pollution is significantly linked to the development of PLC. With the acceleration of industrialization and fossil fuel combustion, pollutants such as fine particulate matter (PM2.5), nitrogen dioxide (NO₂), and black carbon (BC) have become increasingly pervasive [9]. A cohort study reported a positive correlation between these air pollutants and liver cancer risk, with higher levels of PM2.5, NO₂, and BC significantly associated with increased PLC incidence [10]. Beyond PM2.5, accumulating evidence suggests that other environmental pollutants, such as aflatoxins, alcohol, tobacco, and various chemical agents also contribute to liver carcinogenesis [11]. In addition, exposure to heavy metals and volatile compounds (such as arsenic and vinyl chloride), commonly found in industrial emissions, has also been implicated in liver carcinogenesis [12]. A recent ecological study from the United States found that cyanobacterial blooms in drinking water sources were associated with elevated HCC incidence rates, with affected areas showing a 14.2% higher rate compared to non-impacted surface waters [13]. A study conducted in China and Egypt identified a significant association between elevated serum levels of dichlorodiphenyltrichloroethane (DDT), a type of organochlorine pesticide, and increased PLC risk [14, 15]. Collectively, these findings underscore the potentially critical role of environmental pollutants in PLC development and highlight the need for further research.

Bibliometric analysis serves as a powerful tool for evaluating scientific research, providing both quantitative and qualitative insights [16]. It measures research productivity across individuals, institutions, and countries, while also identifying emerging trends and thematic concentrations within a given field [17]. Tools such as CiteSpace and VOSviewer are widely used to visualize scientific collaboration networks and trace the evolution of research themes [18, 19]. This approach has gained wide application in environmental and medical research, exemplified by studies on air pollution and cardiovascular diseases [20], as well as drinking water contamination and cancer [21]. In this study, we apply bibliometric techniques to analyze publications on environmental pollution and PLC from 2000 to 2025. Our analysis systematically explores the current research landscape, identifying major trends, knowledge gaps, and potential future hotspots. To the best of our knowledge, this is the first bibliometric study specifically focused on the intersection between environmental pollution and PLC. The primary aim of this study is not only to provide a comprehensive overview of the existing literature but also to inform future mechanistic research in this field. In addition, these insights can guide policymakers in strengthening environmental regulation and occupational protection and assist clinicians in identifying populations at high risk of PLC.

## Materials and methods

### Data sources and search strategy

**Fig. 1:**
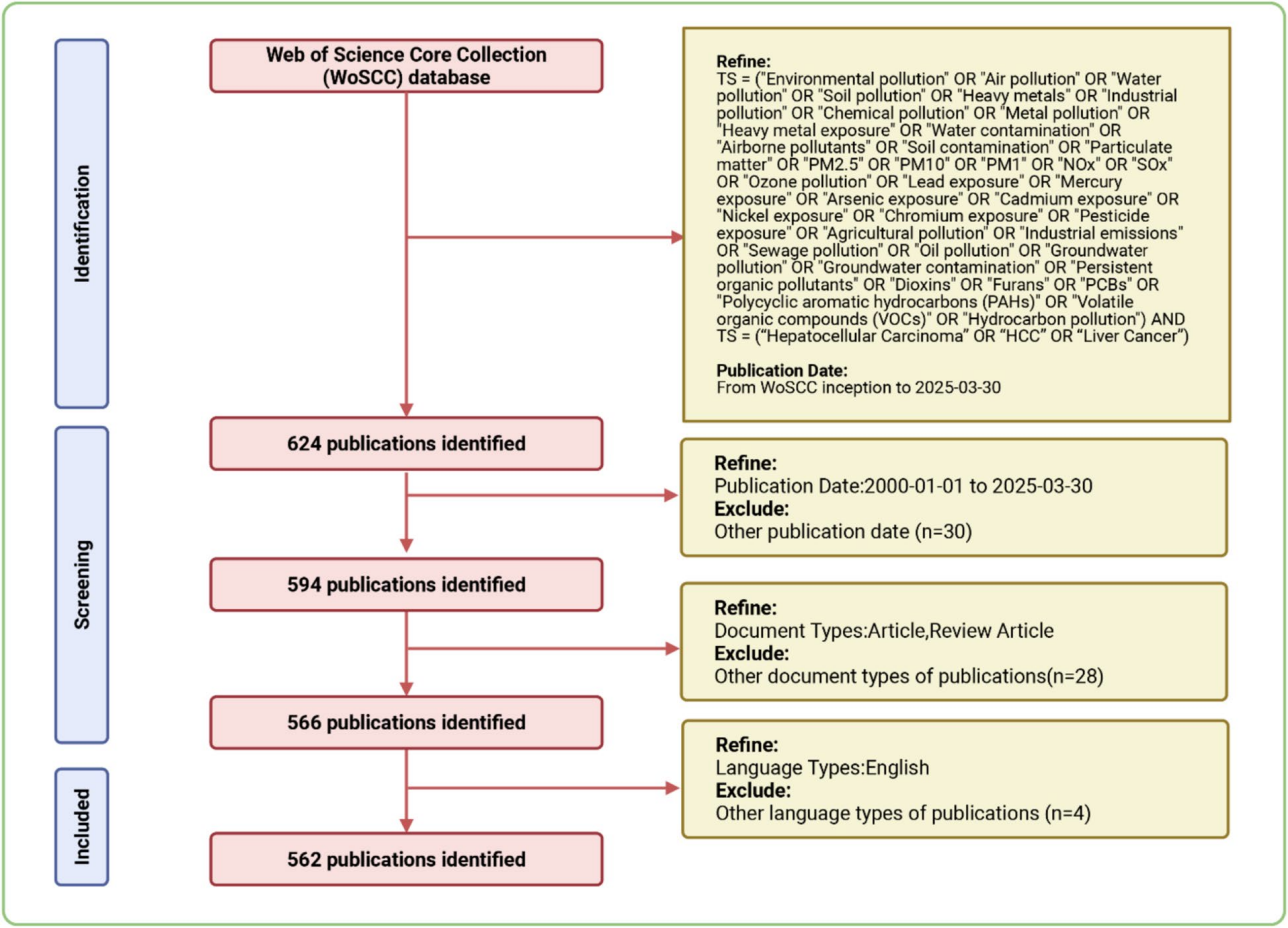
Flowchart of the literature search and selection process

Our raw data were obtained from the Web of Science Core Collection (WoSCC). Recognized as one of the most comprehensive and authoritative databases, Web of Science comprises a vast number of high-quality, high-impact scientific publications across diverse disciplines [22]. The search covered the period from January 1, 2000, to March 30, 2025, and was restricted to English-language publications, including original research articles and reviews. The search strategy was set as follows: (TS = (“Environmental pollution” OR “Air pollution” OR “Water pollution” OR “Soil pollution” OR “Heavy metals” OR “Industrial pollution” OR “Chemical pollution” OR “Metal pollution” OR “Heavy metal exposure” OR “Water contamination” OR “Airborne pollutants” OR “Soil contamination” OR “Particulate matter” OR “PM2.5” OR “PM10” OR “PM1” OR “NOx” OR “Sox” OR “Ozone pollution” OR “Lead exposure” OR “Mercury exposure” OR “Arsenic exposure” OR “Cadmium exposure” OR “Nickel exposure” OR “Chromium exposure” OR “Pesticide exposure” OR “Agricultural pollution” OR “Industrial emissions” OR “Sewage pollution” OR “Oil pollution” OR “Groundwater pollution” OR “Groundwater contamination” OR “Persistent organic pollutants” OR “Dioxins” OR “Furans” OR “PCBs” OR “Polycyclic aromatic hydrocarbons (PAHs)” OR “Volatile organic compounds (VOCs)” OR “Hydrocarbon pollution”) AND TS = (“Liver Cancer” OR “HCC” OR “Hepatocellular Carcinoma”)). Only articles relevant to the study topic and published in English were selected, and all records were downloaded in plain text format for further analysis. The literature retrieval and selection process is illustrated in Fig. 1.

### Data analysis and visualization images

Bibliographic metadata, including titles, authors, keywords, publication years, journals, and citation counts, were extracted for analysis [23]. CiteSpace (v.6.3.R1, Advanced) was employed to construct visual networks of co-authorship, keyword co-occurrence, and co-citation relationships among countries, institutions, authors, and references [24, 25]. The CiteSpace parameters were as follows: time slicing (2000–2025), years per slice (1), node selection (g-index, *k* = 10), and pruning (Minimum Spanning Tree). Modularity *Q* and silhouette scores were used to evaluate the structural validity and clustering reliability of the networks (*Q* > 0.3 indicates significant community structure, and *S* > 0.5 indicates reliable clustering). In the visualizations, node size indicated the frequency of occurrence or number of citations, while color was used to represent different clusters or temporal trends. The width of the connecting lines reflected the strength of association between items such as authors, institutions, or keywords. Microsoft Excel was used to perform basic statistical analyses, such as summarizing annual publication trends. To improve the accuracy of the results, we manually checked and supplemented information on author names, institutional affiliations, journal data, and the labeling of keyword clusters.

## Results

### Annual publication trends

**Fig. 2:**
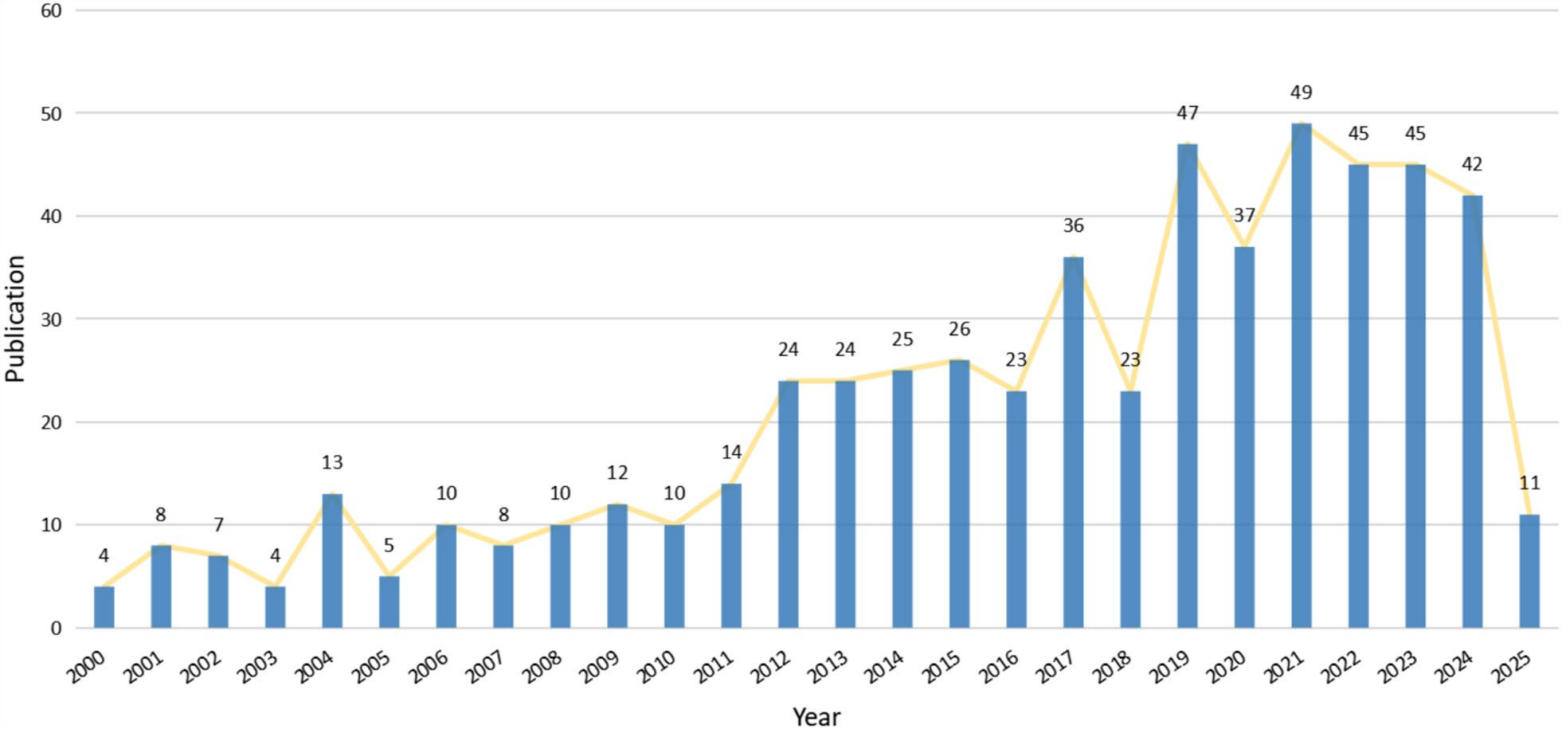
Annual publication trends on environmental pollution and PLC

The annual publication trends related to environmental pollution and PLC are illustrated in Fig. 2. Between 2000 and 2011, the number of publications remained relatively low, averaging fewer than 15 articles per year, with notable fluctuations. From 2012 to 2016, a gradual upward trend was observed, with annual outputs ranging from 20 to 26 articles. This increase likely reflects the emergence of research interest, driven by growing recognition of environmental pollution as a significant risk factor for PLC. Beginning in 2017, the field entered a period of more rapid growth, peaking at 49 articles in 2021, although accompanied by some moderate variability. Between 2021 and 2024, annual publication numbers stabilized at a high level, consistently ranging from 42 to 49 articles. This sustained research momentum suggests that the association between environmental pollution and PLC has become an increasingly prominent focus of scientific investigation.

### Global research collaboration and institutional contributions

**Fig. 3:**
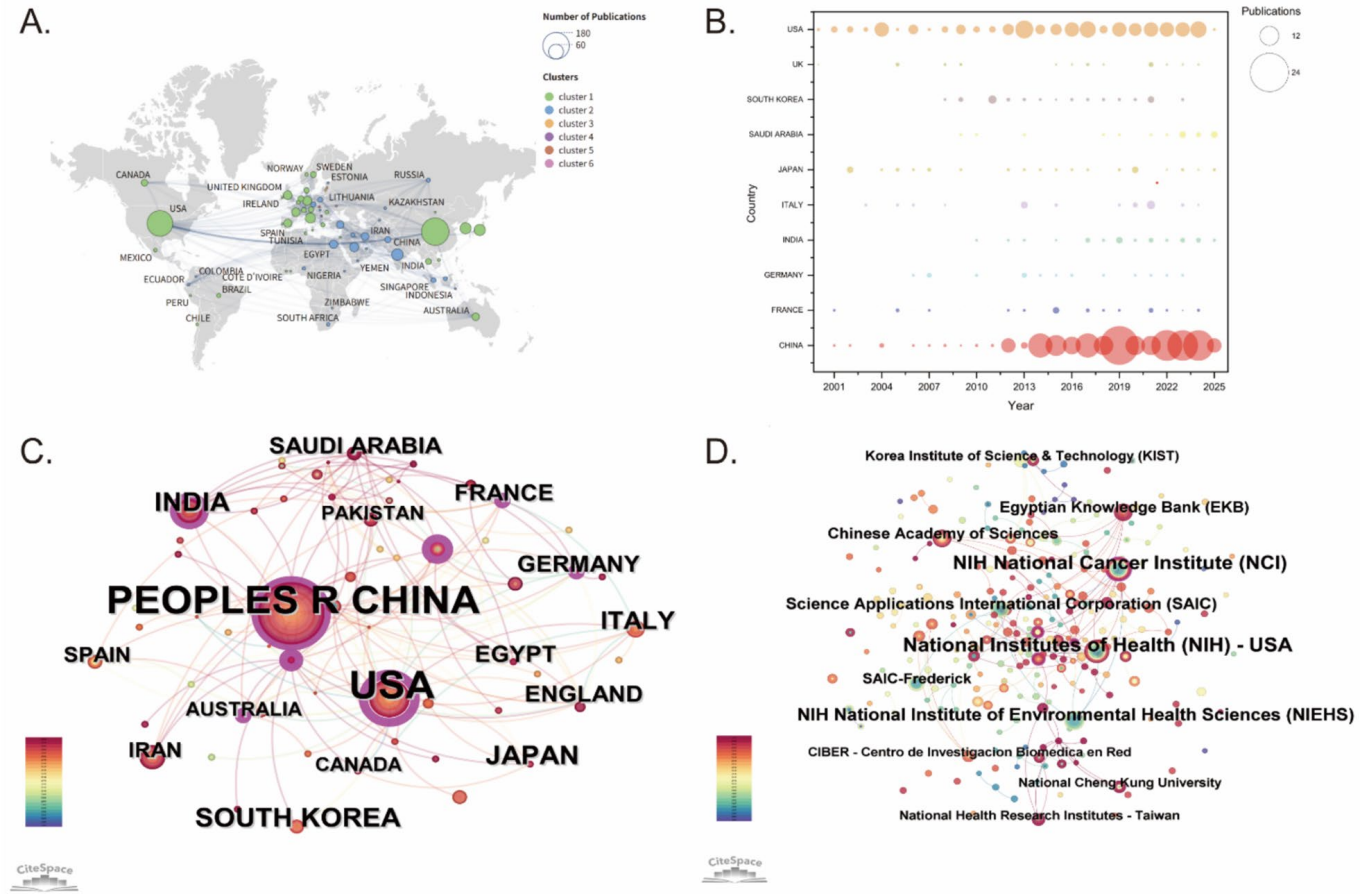
Global collaboration network and institutional contributions in the field of environmental pollution and PLC. **A** Global collaboration map showing international partnerships between countries in a geographic format. **B** Publication trends of the top 10 countries from 2000 to 2025. The size of the circles corresponds to the number of publications from each country, with larger circles representing higher publication numbers in a given year. **C** Country/region co-occurrence network highlighting the frequency of collaborations. **D** Institutional co-occurrence network showing major academic contributors to the field

Figure 3 is an integrated depiction of global collaboration networks, showcasing the major countries/regions and institutions actively contributing to research on environmental pollution and PLC. As shown in Fig. 3A, the international collaboration map reflects widespread global partnerships, with the United States and China emerging as central nodes. This prominence may be attributed to the substantial research investments made by both countries in the fields of environmental health and oncology. These two nations maintain robust academic linkages with countries such as the United Kingdom, Canada, Japan, Spain, Australia, and India. The publication trends of the top 10 contributing countries from 2000 to 2025 are displayed in Fig. 3B. The United States has consistently ranked among the leading contributors, maintaining steady research output over time. Since around 2015, China has demonstrated a substantial increase in publications, emerging as a major force in the field. In addition, countries, such as South Korea, Italy, Japan, and Saudi Arabia have shown steady growth in their contributions, underscoring the growing global interest in the intersection of environmental pollution and PLC. Figure 3C illustrates the co-authorship network among countries and regions, where the United States and China appear as dominant nodes, reflecting their extensive international collaborations. Together, these two nations contribute nearly 44.55% of the total publications. To further characterize international collaboration, we calculated betweenness centrality values (Table 1). Although China leads in output, the United States shows a notably higher betweenness centrality of 0.53, underscoring its pivotal brokerage role in connecting international research clusters. China (0.18) and Germany (0.19) also act as key links within regional networks, highlighting their role in bridging local collaborations. Figure 3D illustrates the institutional co-occurrence network, emphasizing major institutions active in scientific research and academia. Leading institutions such as the National Institutes of Health and the National Cancer Institute (both USA) maintain active partnerships with organizations including Science Applications International Corporation (USA), the Chinese Academy of Sciences (China), and the Egyptian Knowledge Bank (Egypt). The active involvement of international institutions, such as the Korea Institute of Science and Technology (KIST) and Spain’s CIBER, further demonstrates the widespread global engagement in environmental pollution and PLC research. At the institutional level, the National Cancer Institute in the United States shows the highest centrality of 0.11. Several institutions, including the National Institute of Environmental Health Sciences and the Korea Institute of Science and Technology, have a centrality of 0, which may reflect more independent research activity. Table 1 summarizes the leading 10 countries or regions, along with their affiliated institutions, that have made notable contributions to this field of study.

**Table 1:** Top 10 countries and institutions contributing to research on environmental pollution and PLC

Rank	Country	Counts	Centrality	Institution	Counts	Centrality
1	China	176	0.18	National Institutes of Health (USA)	27	0.02
2	USA	155	0.53	National Cancer Institute (USA)	25	0.11
3	India	32	0.13	National Institute of Environmental Health Sciences (USA)	18	0
4	Japan	31	0.03	Science Applications International Corporation (USA)	14	0.02
5	South Korea	30	0.01	Chinese Academy of Sciences (China)	12	0.04
6	Italy	23	0.09	Egyptian Knowledge Bank (Egypt)	12	0.08
7	Saudi Arabia	20	0.09	SAIC-Frederick (USA)	10	0
8	Germany	19	0.19	Korea Institute of Science & Technology (South Korea)	8	0
9	France	17	0.15	CIBER-Centro de Investigacion Biomedica en Red (Spain)	7	0.04
10	England	17	0.09	National Health Research Institutes (Taiwan, China)	7	0.01

### Author co-occurrence and co-citation analysis

**Fig. 4:**
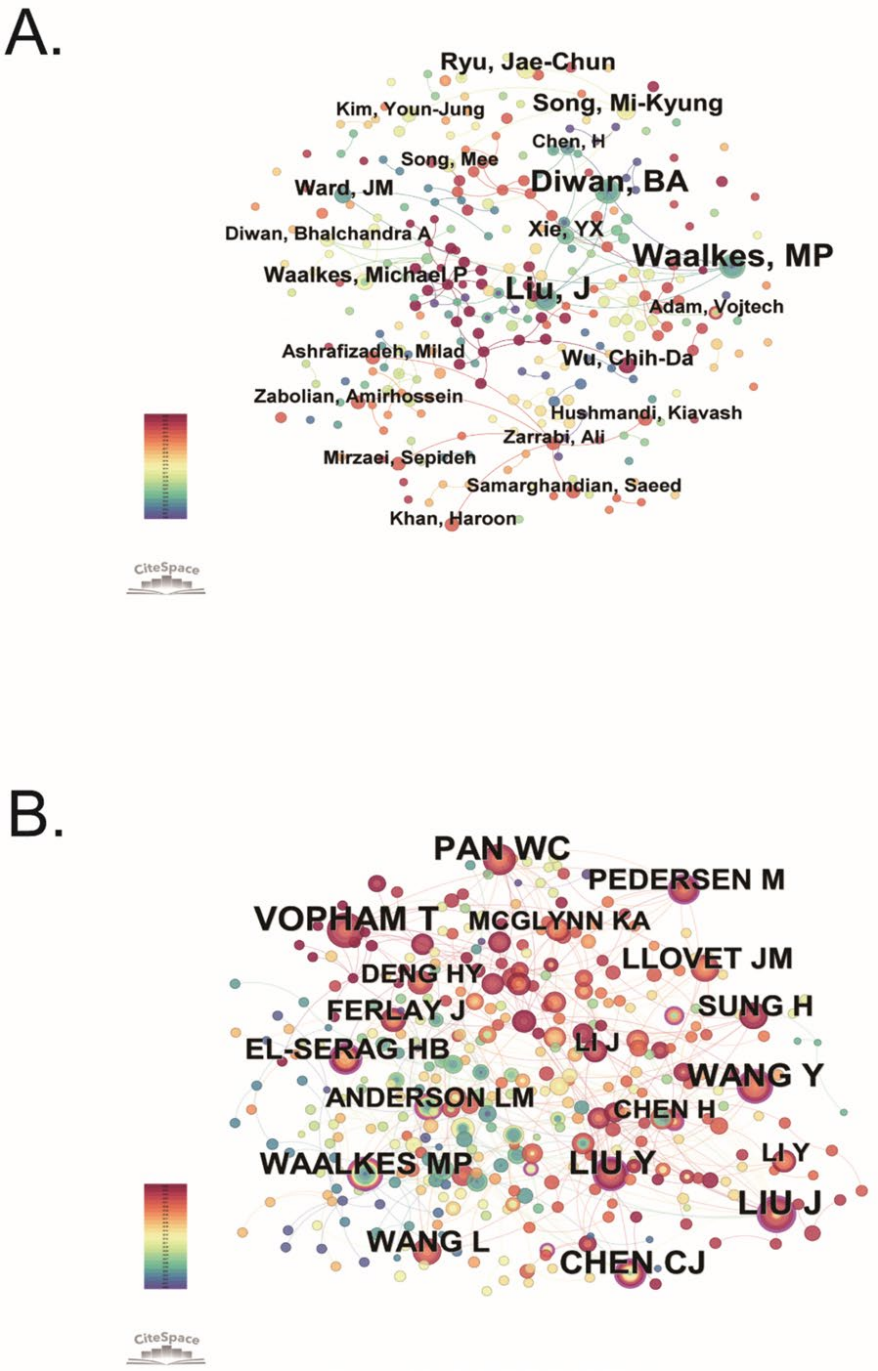
Author co-occurrence and co-citation networks. **A** Author co-occurrence network identifying key researchers and their collaborative relationships. **B** Author co-citation network revealing influential authors frequently cited together in PLC and environmental pollution studies

Figure 4A presents the author co-authorship network, identifying key researchers and their collaborative relationships within the field of environmental pollution and PLC. Notably, Liu Jie, Waalkes MP, and Diwan BA emerge as central figures, indicating their substantial involvement and significant contributions to the field. Active collaboration is observed among several researchers; for example, Waalkes MP frequently collaborates with Ward JM, Liu Jie, and Xie Yuxi. Conversely, Fig. 4B illustrates the co-citation network of authors, emphasizing prominent researchers whose publications are often cited in conjunction by peers within this specific field of study. At the top of this network is Vopham T, followed closely by Liu Jie and Liu Yi, which underscores their foundational roles in shaping the literature on environmental pollution and PLC. Co-citation analysis reflects the intellectual influence and thematic centrality of these authors. Table 2 provides a ranked list of the top 10 authors and co-cited authors contributing to research in this field. These findings highlight the major contributors and their collaborative networks in the field of environmental pollution and PLC. By examining the works of these top authors, readers can trace seminal studies and identify emerging research directions.

**Table 2:** Top 10 authors and co-cited authors on research of environmental pollution and PLC

Rank	Authors	Counts	Co-cited authors	Citations
1	Jie Liu	10	Trang VoPham	31
2	Michael P Waalkes	10	Jun Liu	31
3	Bhalchandra A Diwan	9	Ye Liu	30
4	Mi-Kyung Song	5	Wenchi Pan	29
5	Jae-Chun Ryu	5	Yuan Wang	28
6	Chih-Da Wu	4	Chien-Jen Chen	27
7	Julie M Ward	4	Michael P Waalkes	21
8	Yunxia Xie	4	Lin Wang	19
9	Mirzaei Sepideh	4	Josep M Llovet	19
10	Hao Chen	3	Marie Pedersen	18

### Journal co-citation and dual-map overlay analysis

**Fig. 5:**
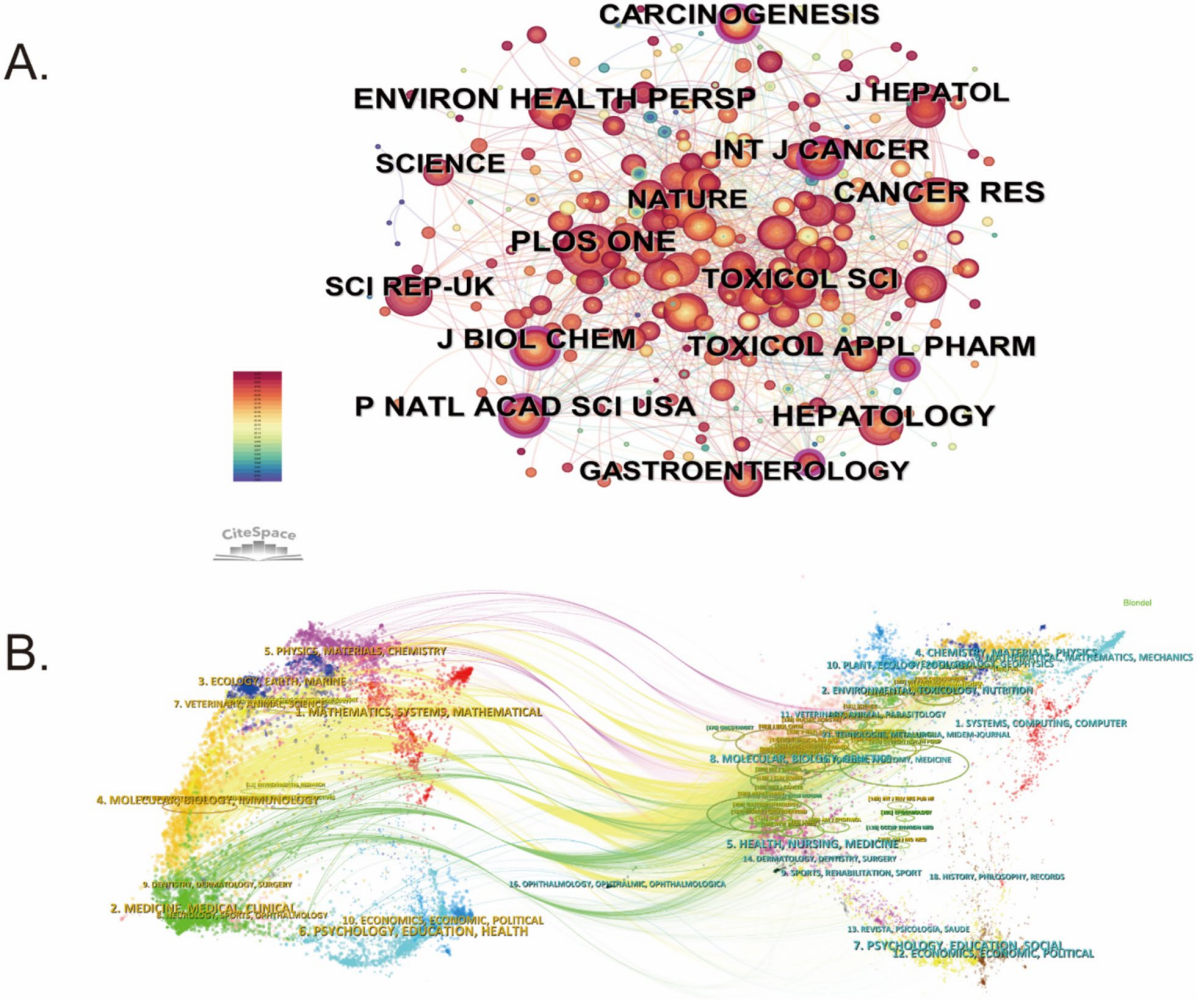
Journal co-citation and dual-map overlay. **A** Journal co-citation network highlighting the most frequently cited journals. **B** Dual-map overlay illustrating citation trajectories between environmental science and clinical medicine journals, emphasizing the interdisciplinary nature of the field

Co-citation refers to the scenario in which two articles are cited together in the reference list of a third article. Table 3 presents the leading 10 journals and co-cited journals in the field of environmental pollution and PLC research. Figure 5A illustrates the journal co-citation network, highlighting the most frequently cited journals in this domain. Journals such as Toxicology and Applied Pharmacology, Environmental Health Perspectives, Hepatology, and Cancer Research emerge as central sources of foundational studies. The Journal of Hepatology boasts the highest impact factor (IF = 26.8), followed closely by Hepatology (IF = 12.9) and Cancer Research (IF = 12.5). These publications serve as pivotal nodes within the co-citation network, underscoring their significant influence and the frequency with which they are cited. Furthermore, journals dedicated to environmental science and public health have emerged as key contributors, highlighting the interdisciplinary character of research that lies at the convergence of environmental pollution and PLC, thereby integrating aspects of environmental and biomedical sciences.

**Table 3:** Top 10 journals and co-cited journals on research of environmental pollution and PLC

Rank	Journal	Counts	IF	JCR	Co-cited journal	Co-citation	IF	JCR
1	Toxicology and Applied Pharmacology	17	3.3	Q2	Environmental Health Perspectives	714	10.1	Q1
2	Science of The Total Environment	14	8.2	Q1	Hepatology	588	12.9	Q1
3	Scientific Reports	13	3.8	Q1	Toxicology and Applied Pharmacology	503	3.3	Q2
4	Ecotoxicology and Environmental Safety	13	6.2	Q1	Cancer Research	490	12.5	Q1
5	Environmental Health Perspectives	12	10.1	Q1	Journal of Biological Chemistry	483	4.0	Q2
6	Environmental Research	11	7.7	Q1	Plos One	455	2.9	Q1
7	Toxicological Sciences	9	3.4	Q2	Toxicological Sciences	437	3.4	Q2
8	International Journal of Cancer	9	5.7	Q1	Carcinogenesis	382	3.3	Q2
9	Toxicology	7	4.8	Q1	Journal of Hepatology	354	26.8	Q1
10	International Journal of Environmental Research and Public Health	7	4.6	Q1	Proceedings of the National Academy of Sciences of the United States	339	9.6	Q1

Figure 5B shows the dual-map overlay of journals, providing a visual summary of how citations flow across various disciplines. On this map, the left side represents citing journals, and the right side depicts cited journals, with connecting lines indicating interdisciplinary citation paths. The citing journals highlight active research frontiers, while the cited journals indicate the foundational work being referenced. The overlay underscores the dominance of disciplines such as “Medicine”, “Clinical”, and “Medical”, along with “Environmental Science”, “Toxicology”, and “Nutrition”. These findings underscore the integrative nature of research in this field, with leading journals across both environmental science and medicine contributing substantially to the development of knowledge on environmental pollution and PLC.

### Reference co-citation, clustering, and citation burst analysis

**Fig. 6:**
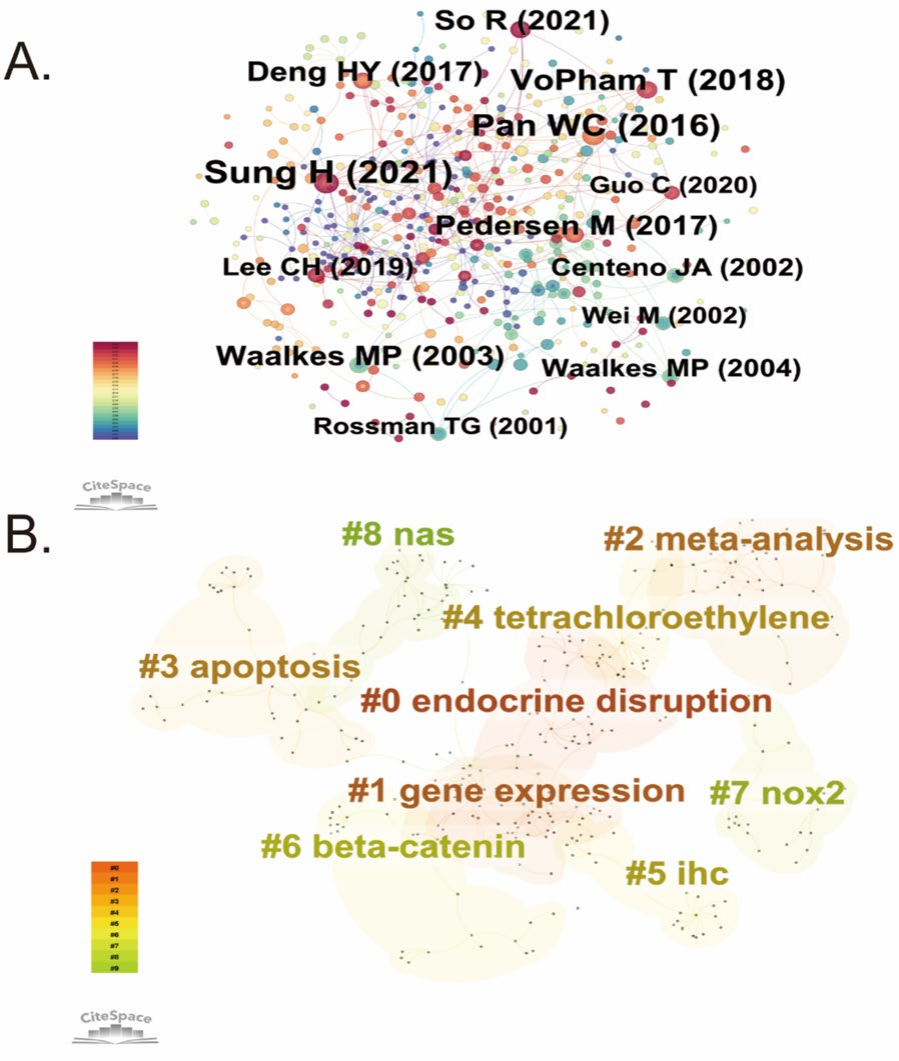
Reference co-citation, clustering, and citation burst analysis in the field of environmental pollution and PLC. **A** Reference co-citation network highlighting influential studies that serve as central nodes in the field. **B** Clustering analysis revealing key research themes

Table 4 presents the ten most co-cited studies in environmental pollution and PLC research. Figure 6A visualizes the co-citation network and highlights key publications such as those by Sung H (2021), Pedersen M (2017), and Pan WC (2016), which occupy central positions in the field. These highly co-cited works have played a pivotal role in advancing the understanding of how environmental pollution contributes to tumor development, particularly PLC. The analysis highlights the most frequently cited references that have significantly influenced this field, serving as foundational studies for ongoing research.

**Table 4:** Top 10 co-cited references on research of environmental pollution and PLC

Rank	Co-cited references	Citations
1	Sung H, 2021, CA Cancer J Clin, v71, p209	17
2	Pan WC, 2016, J Natl Cancer Inst, v108	15
3	VoPham T, 2018, Cancer Causes Control, v29, p563	13
4	Guo C, 2020, CA Cancer J Clin, v70, p313	12
5	Pedersen M, 2017, Environ Res, v154, p226	11
6	Waalkes MP, 2003, Toxicol Appl Pharmacol, v186, p7	11
7	Deng HY, 2017, Int J Cancer, v141, p744	11
8	So R, 2021, Int J Cancer, v149, p1887	11
9	Lee CH, 2019, Int J Environ Res Public Health, v16	8
10	Waalkes MP, 2004, Carcinogenesis, v25, p133	8

Figure 6B demonstrates the cluster analysis of co-cited references, unveiling several prominent thematic areas in this research field. A total of nine distinct clusters are identified, each representing a relatively independent research focus within the broader domain of environmental pollution and PLC risk. The largest cluster, #0 “Endocrine Disruption”, focuses on how endocrine-disrupting chemicals (EDCs) interfere with hormonal regulation and metabolic homeostasis in the liver, underscoring their role in pollution-associated hepatocarcinogenesis. In terms of molecular mechanisms, clusters #1 “Gene Expression”, #3 “Apoptosis”, #6 “β-catenin”, and #7 “NOX2” reflect sustained interest in pollutant-induced tumor promotion, encompassing transcriptional regulation, cell death pathways, oncogenic signaling such as Wnt/β-catenin, and oxidative stress mediated by NADPH oxidase 2. Regarding pollutant-specific themes, cluster #4 “Tetrachloroethylene” highlights chemical compound exposure, particularly its hepatotoxicity and carcinogenicity, emphasizing its role in the environmental etiology of PLC. Cluster #5 “IHC” denotes the routine use of immunohistochemical techniques for tissue-level detection of liver cancer-related markers. In terms of methodology, cluster #2 “meta-analysis” reflects the increasing application of systematic reviews and meta-analyses, indicating their growing value in evidence synthesis in this field. Additionally, cluster #8 “NAS” refers to publications by authoritative institutions, such as the National Academy of Sciences (USA), marking a transition toward standardized research and integration into public health policy frameworks. This cluster analysis illustrates the thematic diversity in this field, encompassing mechanisms of pollutant-induced carcinogenesis, biomarker detection methods, and evidence-based evaluations supported by institutional data. The interdisciplinary nature of these clusters demonstrates that research on environmental pollution and PLC is evolving toward a comprehensive framework that integrates basic scientific inquiry with public health response.

**Fig. 7:**
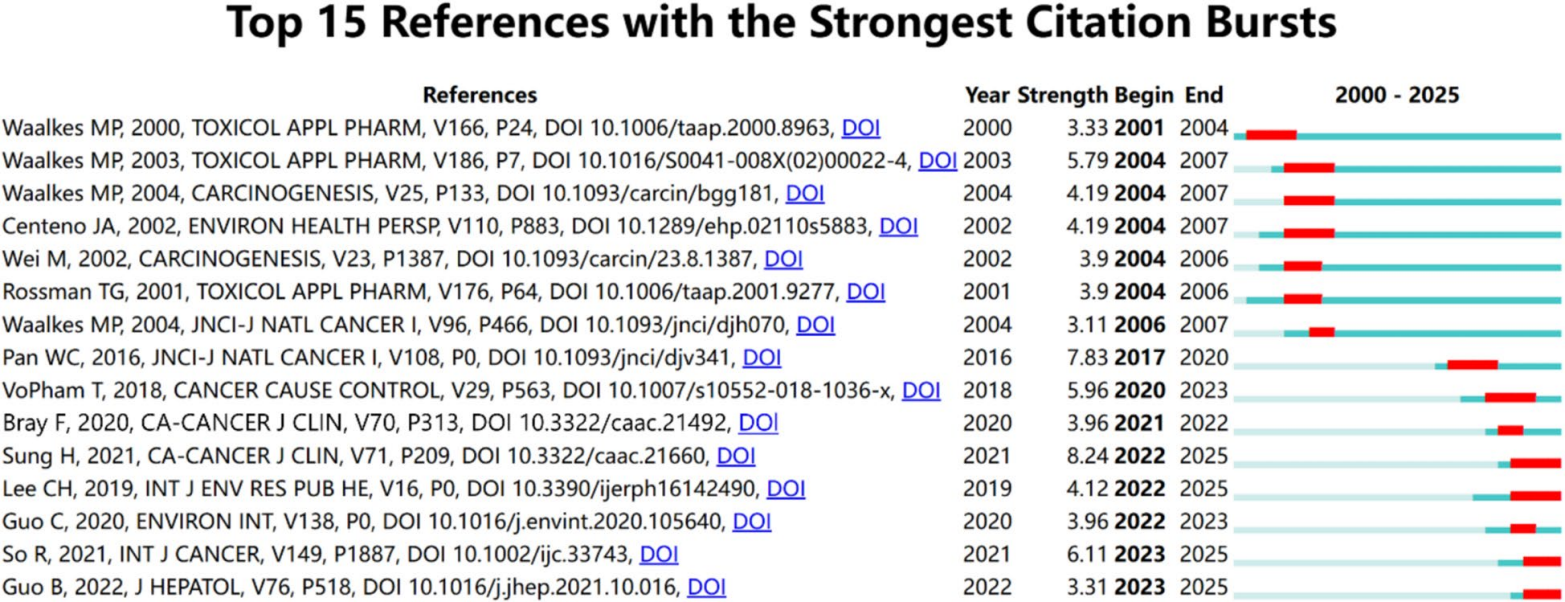
Citation burst analysis identifying the top 15 references with the strongest citation bursts

Figure 7 identifies the 15 references with the most pronounced citation bursts, reflecting studies that saw a notable rise in citations during certain periods. In bibliometrics, citation bursts point to references that gain significant and sustained attention over a short span, indicating their impact on scholarly discourse. The burst strength of these 15 references ranged from 3.11 to 8.24, typically spanning two to four years. Among them, a notable study conducted by Waalkes MP in 2004 using a murine model demonstrated that fetal exposure to inorganic arsenic could lead to HCC in adult male mice. This work provided early experimental evidence linking heavy metal exposure to liver carcinogenesis. Another influential study by Rina So et al. (2021), based on six prospective European cohorts, reported that ambient air pollution, particularly PM2.5 and NO₂, was significantly and positively associated with liver cancer risk. Importantly, this association remained robust even at pollutant concentrations below current EU regulatory limits. Taken together, these representative studies highlight a clear evolution in research priorities: from early toxicological experiments elucidating potential carcinogenicity to epidemiological investigations assessing real-world population risks.

### Keyword co-occurrence, clustering, temporal, and burst analysis

**Fig. 8:**
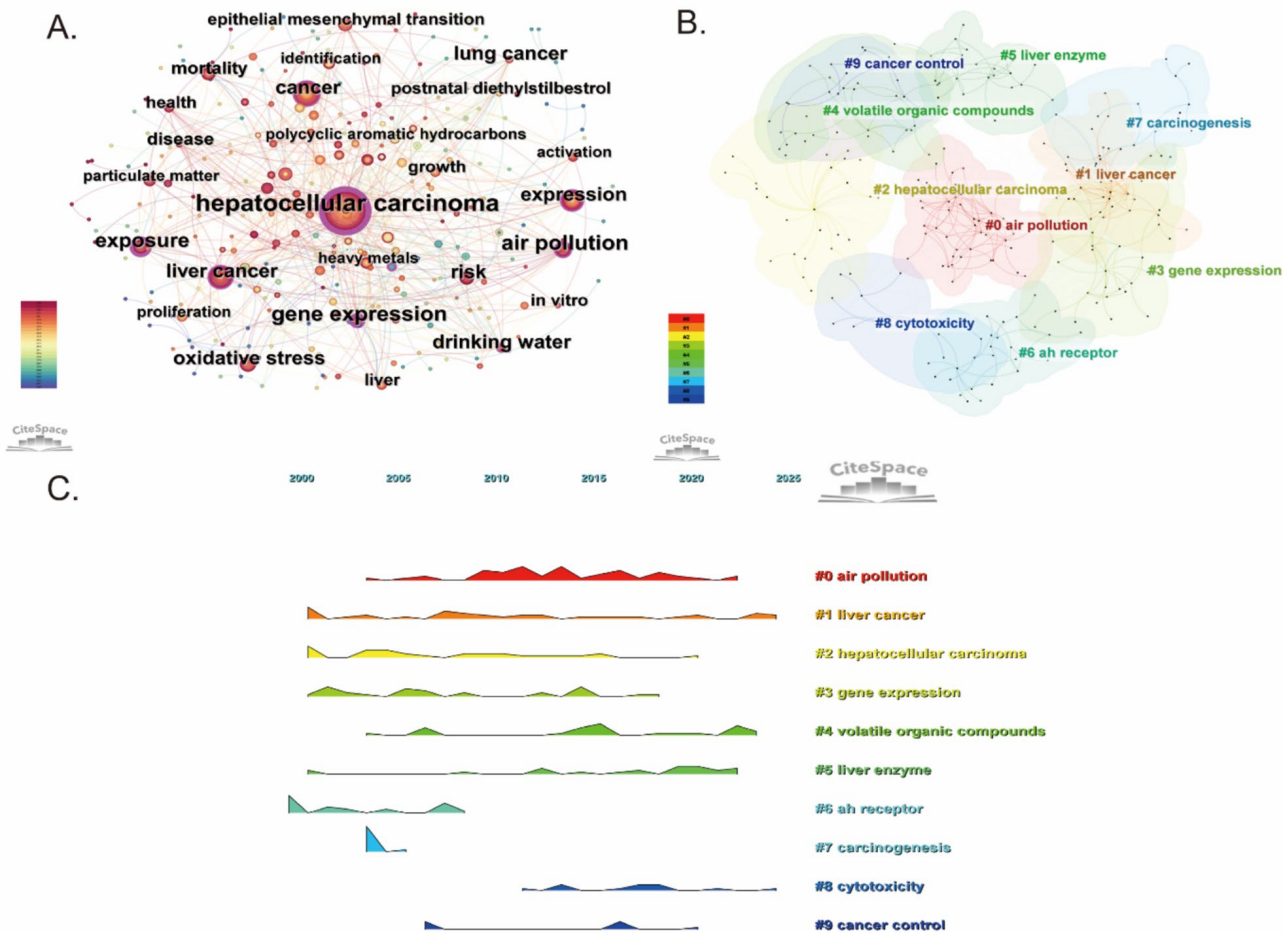
Keyword co-occurrence, clustering, and temporal analysis in the field of environmental pollution and PLC. **A** Keyword co-occurrence network revealing frequently used terms. **B** Keyword clustering analysis identifying distinct research themes. **C** Temporal trends of keyword clusters illustrating the evolution of research themes over time

Figure 8A illustrates the keyword co-occurrence network, highlighting the most frequently used terms in environmental pollution and PLC research. The most prominent terms include “hepatocellular carcinoma”, “liver cancer”, “air pollution”, “exposure”, “particulate matter” and “heavy metals” indicating these are central topics in this research domain. The figure also reveals that mechanism-related terms such as “oxidative stress”, “gene expression”, “proliferation”, and “epithelial mesenchymal transition”, which emphasize the focus of current research on the molecular links between environmental pollution and liver cancer. The high density of connections among the keywords in the figure suggests significant crossover and integration among the research themes.

Figure 8B illustrates the keyword clustering map generated by CiteSpace. To assess the validity of the clustering results, we applied two standard bibliometric metrics: modularity *Q* and the silhouette score. The modularity *Q* value was 0.5351, and the average silhouette coefficient reached 0.8439, indicating that the clusters are clearly defined and of high quality. When the modularity *Q* value exceeds 0.3, it indicates that the community structure is meaningful, while a silhouette score above 0.5 confirms the reliability of the clustering results. Cluster #0 “air pollution” and Cluster #4 “volatile organic compounds” represent two major sources of environmental pollution, corresponding to atmospheric and chemical exposures. These are the types of pollutants most strongly associated with liver cancer risk in this study. Cluster #1 “liver cancer” and Cluster #2 “hepatocellular carcinoma” focus on disease-centered research themes and serve as the conceptual link between environmental exposure and health outcome evaluation. Cluster #3 “Gene Expression”, Cluster #6 “Ah receptor”, and Cluster #7 “carcinogenesis” reflect key molecular mechanisms involved in environmental pollution-induced hepatocellular carcinoma, including transcriptional regulation and signal transduction pathways. Cluster #5 “liver enzyme” and Cluster #8 “cytotoxicity” highlight the cellular-level damage caused by environmental pollutants, typically assessed through liver function markers and cytotoxicity assays. Cluster #9 “cancer control” focuses on translating scientific evidence into public health strategies, reflecting the growing emphasis on bridging environmental health research with policy implementation.

Figure 8C presents the temporal trends of keyword clusters using a mountain-shaped timeline graph, which illustrates the evolution of research themes over time. Cluster #0 “air pollution” and Cluster #1 “liver cancer” remained consistently prominent throughout the study period, underscoring their central role in the field. Research associated with Cluster #3 “gene expression” and Cluster #6 “Ah receptor” peaked between 2005 and 2015; during this period, scholars increasingly investigated the mechanistic pathways linking environmental pollutants to hepatocarcinogenesis, coinciding with the rapid development of molecular biology techniques. The subsequent decline may reflect a relative saturation of these studies, with attention shifting toward novel approaches such as exposomics and population-based risk assessments. In contrast, Cluster #4 “volatile organic compounds” has shown a marked upward trend in recent years, reflecting growing awareness that various environmental pollutants, including VOCs, possess potential hepatocarcinogenic effects. The timeline analysis highlights both the evolution of key research themes and their growing connection with cancer prevention and control strategies.

**Fig. 9:**
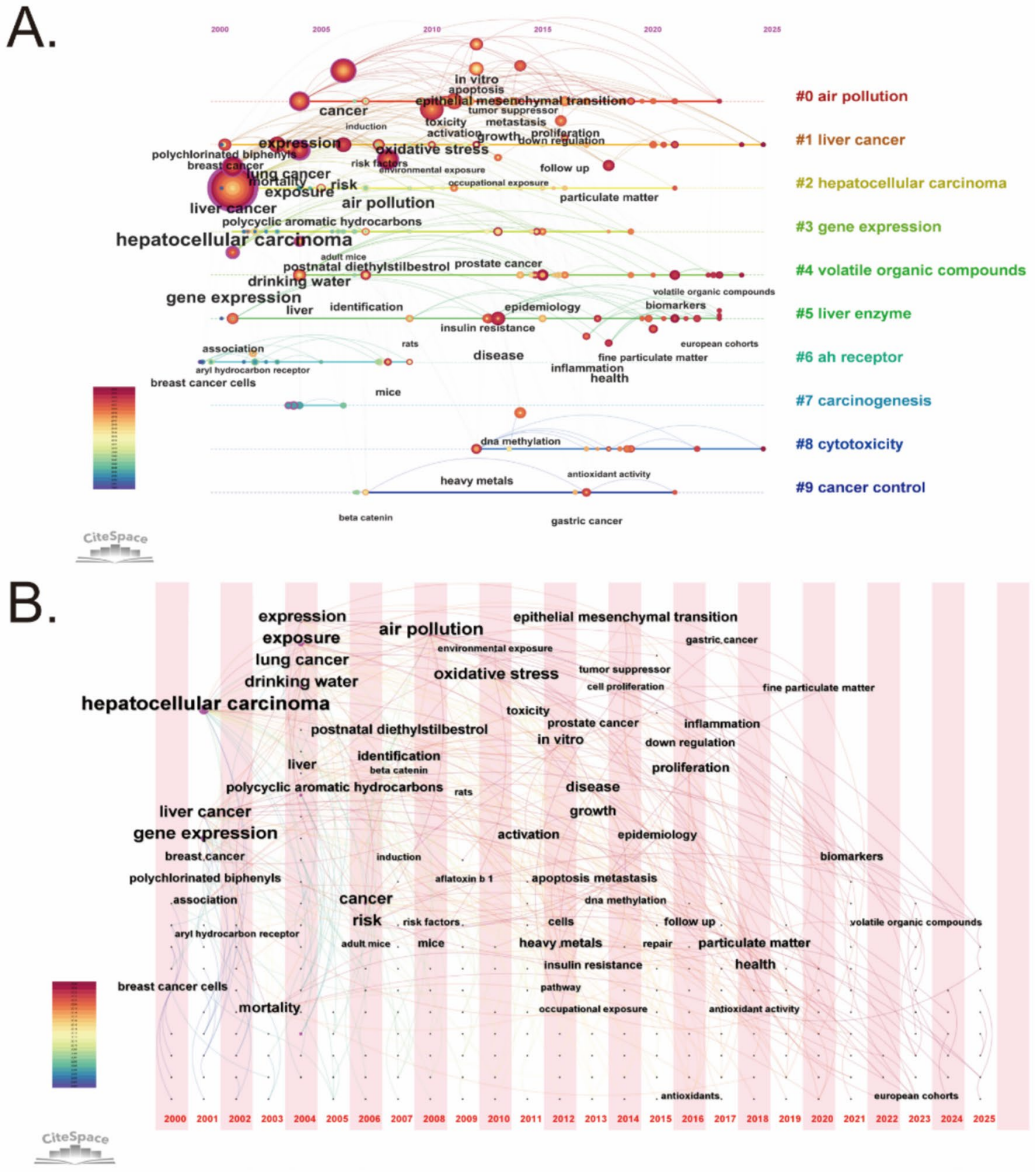
Keyword timeline and time-zone map. **A** Timeline of keyword usage illustrating the evolution of key topics from 2000 to 2025. The size of the nodes reflects the frequency of occurrence of different research directions over the past 25 years, with larger nodes representing a higher frequency of occurrence. **B** Time-zone map depicting the development and progression of research themes over time. The location of each keyword in the map represents the year when it first became active in related scientific research application fields. The larger the size of the keyword, the higher its co-occurrence in the field. These visualizations not only depict the historical progression of research themes but also highlight emerging hotspots and provide guidance for future research priorities

Figure 9A depicts how keyword usage has progressed over time in studies related to environmental pollution and PLC, underscoring topic evolution. Keywords such as “air pollution”, “exposure”, “mortality rate”, “liver cancer”, and “hepatocellular carcinoma” have consistently appeared since 2000, reflecting the academic community’s sustained interest in the link between environmental pollution and the development of PLC. In recent years, the increasing frequency of terms like “volatile organic compounds”, “heavy metals”, “drinking water”, “biomarkers”, “epidemiology”, and “follow-up” suggests a growing emphasis on accurately assessing health risks related to specific pollutant sources and conducting epidemiological studies that incorporate long-term follow-up. Figure 9B presents the time-zone map, offering a broader view of the temporal development of research themes. During the early phase from 2000 to 2010, keywords such as “air pollution”, “exposure”, “liver cancer”, and “mortality” were frequently used. This reflects a growing recognition of air pollution as a potential risk factor for PLC, during which many countries initiated epidemiological monitoring of environmental exposures such as air pollution and gradually implemented national environmental quality standards. Around 2010, keywords including “oxidative stress”, “apoptosis”, “DNA methylation”, and “epithelial mesenchymal transition” became more prominent. During this period, research focus shifted to elucidating specific mechanisms linking environmental pollution to liver cancer, possibly driven by advances in exposomics and epigenetic technologies. Since 2018, the continued emergence of keywords like “biomarkers”, “volatile organic compounds”, “drinking water”, “particulate matter”, “follow-up”, and “epidemiology” marks a transition toward evaluating the health risks of specific pollutants. This stage reflects a growing emphasis on translating these findings into population-level interventions, often combining clinical studies and public health policy measures. Overall, Fig. 9 not only presents the historical evolution of research focuses in the field, but also helps identify emerging hotspots in recent years, providing valuable guidance for future research.

**Fig. 10:**
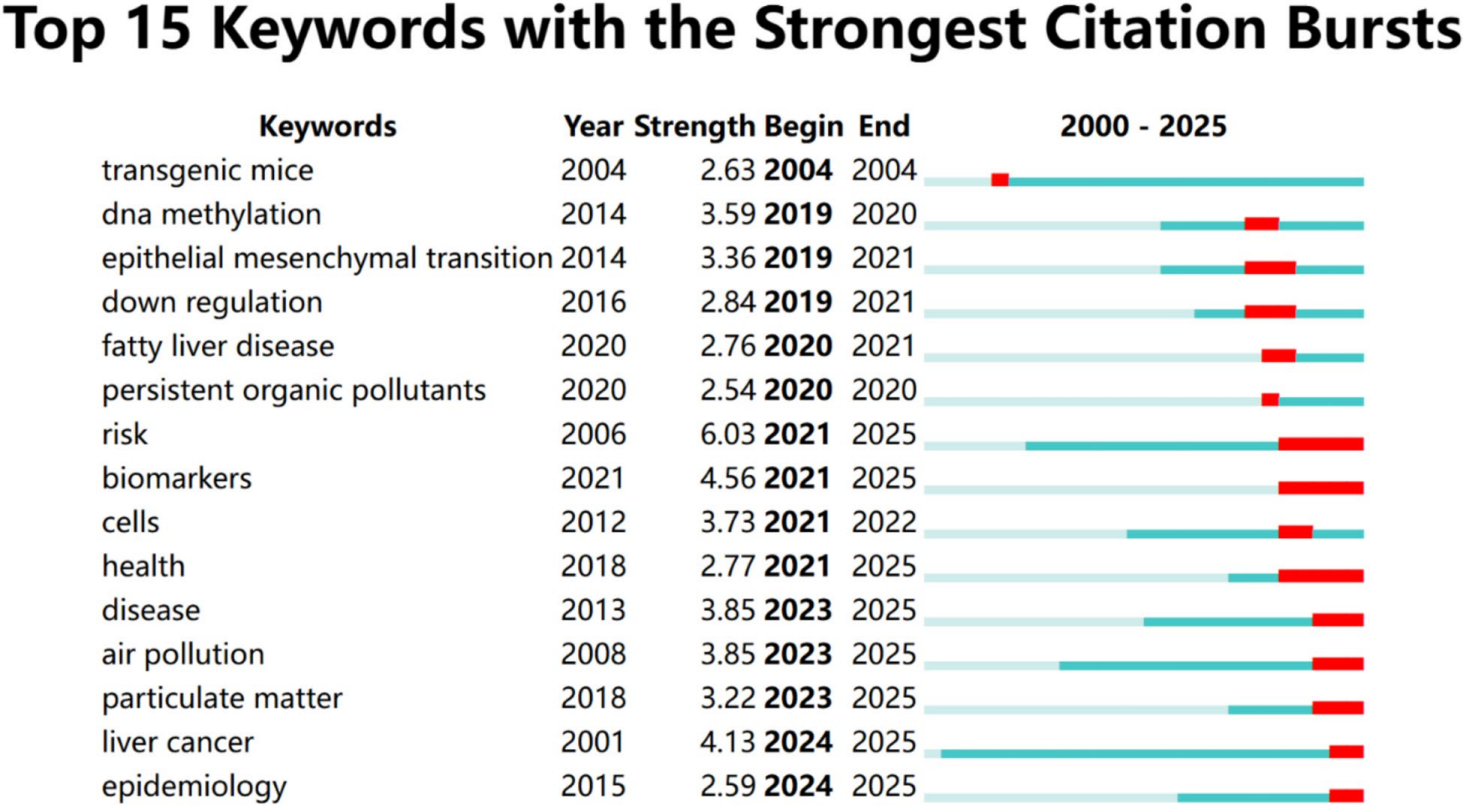
Top 15 keywords with the strongest citation bursts

Figure 10 showcases the top 15 keywords with rapid citation increases, indicating significant changes in research attention over the years. The earliest burst was observed for “transgenic mice” in 2004, indicating that early investigations primarily used animal models to explore the toxicological mechanisms of environmental pollutants. In the following phase, keywords such as “DNA methylation” (2019–2020), “epithelial mesenchymal transition” (2019–2021), and “down regulation” (2019–2021) became prominent, signaling increased attention to the epigenetic and cellular-level pathways through which pollutants may influence hepatocarcinogenesis. From 2020 onward, research interest shifted toward specific pollutant types. This is reflected in the emergence of keywords like “persistent organic pollutants” (2020), “particulate matter” (2023–2025), and “air pollution” (2023–2025), emphasizing the growing focus on defined environmental hazards and their links to PLC. In the most recent phase, keywords such as “risk” (2021–2025), “health” (2021–2025), and “epidemiology” (2024–2025) have appeared more frequently, indicating a transition toward population-level studies and public health strategies. Taken together, these trends suggest that the field is gradually moving from mechanistic exploration toward the development of prevention and intervention approaches for PLC in public health contexts.

## Discussion

This study presents the first comprehensive bibliometric analysis of global research on environmental pollution and PLC from 2000 to 2025. The annual publication trend indicates that although early research output was relatively limited, a significant increase has occurred since 2017, with the number of publications remaining consistently high in recent years. This sustained growth reflects increasing scholarly recognition of environmental pollution as a critical and emerging risk factor for PLC. The analysis of country-level, institutional, and author co-occurrence networks demonstrates that China and the United States have become major contributors and central nodes in international collaboration within this field. Additionally, countries such as South Korea, Japan, and Italy have shown a rising level of engagement, highlighting the global expansion of interest in environmental determinants of liver cancer. Collaborations among leading institutions emphasize the interdisciplinary nature of this study. Keyword co-occurrence and clustering analyses reveal a broad and evolving research landscape. Over time, research topics have expanded from basic mechanistic studies to investigations focusing on specific pollutants and their impact on public health. Notably, keywords such as “air pollution”, “particulate matter”, “liver cancer”, and “hepatocellular carcinoma” have consistently appeared at the core of the literature over the past two decades.

Furthermore, cluster analysis shows that current research focuses primarily on two areas: molecular mechanisms and exposure assessment. The frequent appearance of keywords such as “oxidative stress”, “gene expression”, and “Ah receptor” indicates sustained interest in how pollutants contribute to liver cancer through oxidative stress, signaling pathway activation, and transcriptional regulation. In addition, the increasing use of terms like “follow-up” and “epidemiology” suggests a growing shift toward research on clinical risk monitoring and public health interventions at the population level. However, as the understanding of liver cancer pathogenesis has deepened, researchers have increasingly recognized the multifactorial and complex nature of its causes. In particular, environmental pollution and exposure have gained growing attention as significant yet previously underexplored carcinogenic risk factors [26].

Previous bibliometric studies have examined the impact of environmental pollution on a wide range of diseases from various perspectives, offering important insights into the development of this research field. Among these studies, air pollution has emerged as a key area of concern. For instance, Shu et al. analyzed research trends related to PM2.5 and asthma using bibliometric and visualization techniques, reflecting the academic community’s sustained interest in the health effects of air pollution [27]. Another study investigated the association between long-term exposure to fine particulate matter and cancer risk, highlighting growing attention to air pollution as a potential carcinogen [28]. In addition, bibliometric analyses have explored the relationship between waterborne pollutants and cancers such as bladder cancer, as well as the link between occupational pesticide exposure and tumorigenesis [21, 29]. These findings collectively suggest that environmental pollutants play a significant role in cancer development. Despite these advances, there remains a lack of systematic bibliometric analysis specifically addressing the link between environmental pollution and PLC. This study fills the existing gap by offering a global synthesis of the research landscape and emerging trends within this interdisciplinary domain. The liver is the primary organ responsible for the detoxification and metabolism of environmental chemicals [30]. These chemicals can enter the human body through three main routes: inhalation, ingestion, and dermal absorption [31]. Inhaled pollutants such as PM2.5 and benzene may deposit in the respiratory tract and subsequently enter the systemic circulation, eventually reaching the liver and triggering oxidative stress and chronic inflammation [32]. Ingestion of contaminated food and water represents another important pathway, involving substances such as aflatoxins and heavy metals, which are absorbed through the gastrointestinal tract and transported to the liver via the portal vein [33]. Dermal absorption is particularly relevant for lipophilic or low-molecular-weight chemicals, including certain pesticides and phthalates [33]. After crossing the skin barrier, these compounds enter systemic circulation. Once in circulation, they accumulate in the liver and promote oxidative stress, inflammation, and genotoxic damage [28], processes that contribute to the progression of PLC.

Numerous studies have demonstrated the impact of ambient PM2.5 exposure on the risk of developing liver cancer. A multi-center study conducted in Europe reported statistically significant hazard ratios for several elemental components of PM2.5, with sulfur and vanadium showing the strongest associations [8]. In the United States, an ecological study found that higher ambient PM2.5 concentrations were correlated with an increased incidence of PLC [29]. Similarly, a prospective cohort study in Taiwan reported that residential exposure to elevated levels of PM2.5 was associated with a higher risk of HCC [30]. Mechanistically, these pollutants can trigger oxidative stress, chronic inflammation, and genotoxic effects [32]. These effects damage hepatocytes, induce DNA mutations, and ultimately promote tumor development [33].

Building on this mechanistic understanding, our bibliometric findings can be further interpreted through the lenses of the exposome, eco-social theory, and translational public health. The exposome encompasses the totality of lifelong external exposures and their internal biological responses [34]. From this perspective, PLC risk can be viewed as the cumulative result of lifelong environmental insults. Our results highlight air pollution, particulate matter, heavy metals, and VOCs as key contributors to PLC. For instance, long-term consumption of arsenic-contaminated water has been shown to increase PLC risk in a dose-dependent manner [35], while another study found significantly higher blood cadmium levels in PLC patients compared to chronic hepatitis patients and controls [36]. These examples illustrate how cumulative exposures shape liver cancer risk and provide a useful framework for interpreting bibliometric evidence.

From an eco-social perspective, PLC risk emerges from the interplay of social structures, ecological environments, and biological susceptibility. Occupational exposures exemplify this interaction [37]. Workers in petrochemical, metallurgical, and chemical industries are frequently exposed to organic solvents, heavy metals, and polycyclic aromatic hydrocarbons, facing elevated PLC risks [26]. Moreover, individuals with underlying liver conditions, such as chronic hepatitis B virus infection or MASLD, may experience synergistic effects when exposed to pollutants. Evidence from a prospective cohort study in Taiwan demonstrated that PM2.5 exposure in combination with HBsAg positivity significantly accelerated PLC development [38]. This suggests that occupational and environmental toxicant exposures not only act as independent carcinogens, but may also exacerbate the progression of metabolic dysfunction-associated fatty liver disease toward PLC.

From a translational public health perspective, our study provides several implications for policymakers. First, the findings emphasize the need for stricter environmental regulations and stronger occupational protections, particularly for air pollutants and industrial chemicals. Enhanced air quality monitoring and increased use of renewable energy should be prioritized in heavily polluted areas. Second, expanding early PLC screening programs for high-risk populations, including patients with chronic liver disease and workers with occupational exposures, could improve timely detection and intervention. Finally, public education campaigns are essential to raise awareness of the link between environmental pollution and PLC, promote behavioral change, and strengthen prevention efforts at the societal level.

Our bibliometric results further indicate that since 2018, research has increasingly focused on biomarkers, VOCs, drinking water contaminants, particulate matter, and long-term epidemiological follow-up, reflecting a shift toward integrating environmental exposures with both molecular mechanisms and population-level assessments. However, evidence on VOC-related hepatocarcinogenesis remains fragmented, studies on drinking water contaminants are geographically limited, and the carcinogenic mechanisms of particulate matter are not yet fully elucidated. Moreover, the lack of large-scale prospective cohort studies restricts validation of these associations in diverse populations. Future research should prioritize addressing these gaps through longitudinal cohort designs and multi-omics approaches to clarify the pathways by which pollutants drive hepatocarcinogenesis. Such efforts will provide a scientific foundation for effective public health interventions, ultimately reducing the global burden of PLC.

In conclusion, our research not only maps the current landscape of studies on environmental pollution and PLC, but also highlights priority directions for future investigation. These findings provide guidance for mechanistic research, population-level assessments, and targeted public health interventions.

## Limitations

Firstly, this study relied exclusively on publications indexed in the Web of Science. Although this database substantially overlaps with other major sources such as PubMed and Scopus, it may still exclude some regional or specialized journals. As a result, certain relevant studies might have been overlooked. Future research should consider using multiple databases to provide a more comprehensive view of the literature. Secondly, bibliometric indicators such as citation counts do not necessarily reflect the true clinical or policy impact of research. Citation frequency can be affected by journal reputation and network factors rather than the intrinsic value or real-world applicability of a study. Therefore, qualitative evaluations should supplement bibliometric findings. Third, despite our careful analysis, our findings primarily rely on the automated bibliometric outputs generated by CiteSpace. This dependency may limit the depth of our insights. Future research should combine automated analysis with manual verification and multiple tools to enhance rigor. Finally, our analysis relied on raw citation counts without applying normalized indicators such as the mean normalized citation score (MNCS) or the field-weighted citation impact (FWCI). This limitation may reduce the robustness of cross-field comparisons, and future studies should incorporate normalized metrics to improve interpretability across disciplines.

## Conclusions

This study provides a comprehensive bibliometric overview of global research on the association between environmental pollution and PLC from 2000 to 2025. The findings reveal a clear thematic evolution, from early studies focused on toxicological mechanisms to more recent research examining specific pollutants, including air pollution and heavy metals, and their impact on liver cancer risk. Population risk assessment and long-term epidemiological follow-up have gained increasing attention. Overall, these results underscore the growing recognition of environmental pollution as a significant contributor to liver cancer and highlight the importance of translating research insights into effective public health strategies to mitigate PLC risk and improve global health outcomes.

## Data Availability

The datasets generated and analyzed during the current study are available from the WoS Core Collection database. Any additional materials or information related to this study can be obtained from the corresponding author upon reasonable request.
